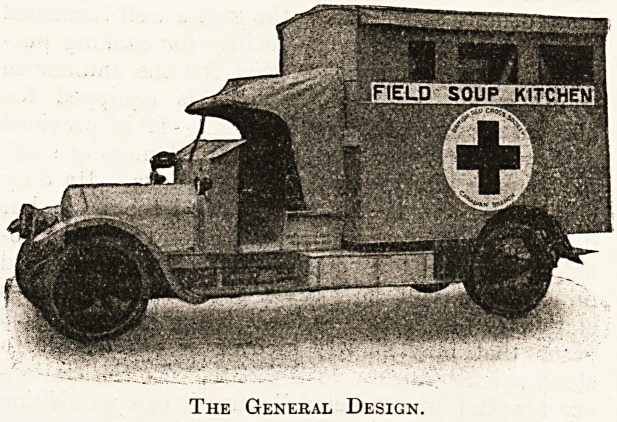# A Red Cross Field Soup Kitchen: The Design and Equipment in Detail

**Published:** 1915-04-17

**Authors:** 


### A RED CROSS FIELD SOUP KITCHEN.

### The Design and Equipment in Detail.

(FROM A CORRESPONDENT.)

In the preceding article Sir George Beatson points out that in the case of the motor-ambulance the feeding arrangements are of a more or less improvised kind, unless it happens to form one of the fleet or convoy, when, he adds, "there usually accompanies the unit a well-furnished motor-kitchen with every facility for cooking purposes." It is, therefore, interesting to supplement his general account by a description of one of the motor field-kitchens, and for that purpose no better example could be chosen than the one presented to the Canadian Red Cross Society, which accompanies the Dominion's contingent at the Front.

The Field Soup Kitchen, presented by Major Leonard to the Canadian Red Cross Society for use with the Canadian contingent at the Front, deserves special notice as a type of the means by which comfort is organised for transport purposes in the field. Of the many motor conveniences for field service this soup kitchen is one of the most interesting. It is surprising how scientific a system is carried so compactly on this Wolseley 16-20 h.p. chassis, which is of the standard ambulance type as supplied to the British War Office and British Red Cross Society. Not only is the water used filtered but also sterilised, for such a process is important

The Kitchen Equipment.

when it is remembered that the supply must often be obtained from any convenient stream or pond.

The Berkefeld filter employed is one of the high capacity type, so that a considerable volume of water may be pumped through it into the steriliser in a reasonable time, and for this purpose a semi-rotary pump and twenty feet of hose-pipe are fitted.

The steriliser has a capacity of approximately forty gallons. The heating is effected by a carefully arranged system of "Primus" type burners, which are fed from a large reservoir of paraffin placed under the urns. The reservoir holds sufficient fuel to last for several days. There are three urns, each having a capacity of some five gallons. These are so arranged as to make it possible for three different

The General Design.

types of hot drinks to be made at one time, and so soup, Bovril, and coffee or other beverage can all be served. A constant supply of no less than 880 half-pints of hot liquid is estimated to produce 7,040 cups of half-pint capacity in a twenty-four hour day. That is to say this one field kitchen can suffice for seven battalions. Minor points of interest in connection with the urns are that they cannot be run dry as the taps are raised. The urns themselves are copper-jacketed and porcelain-lined. The price of the car fully equipped is £585.

## Figures and Tables

**Figure f1:**
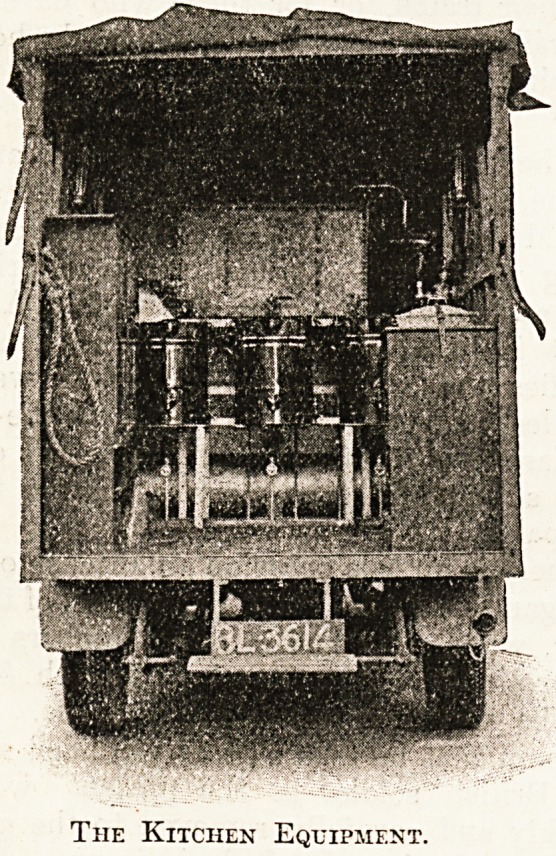


**Figure f2:**